# A core-genome multilocus sequence typing scheme for the detection of genetically related *Streptococcus pyogenes* clusters

**DOI:** 10.1128/jcm.00558-23

**Published:** 2023-10-10

**Authors:** Myrthe M. A. Toorop, Margriet E. M. Kraakman, Irene V. Hoogendijk, Joffrey van Prehn, Eric C. J. Claas, Els Wessels, Stefan A. Boers

**Affiliations:** 1 Department of Medical Microbiology, Leiden University Medical Center, Leiden, the Netherlands; Westfalische Wilhelms-Universitat Munster, Münster, Germany

**Keywords:** core-genome multilocus sequence typing, cgMLST, next-generation sequencing, NGS, *Streptococcus pyogenes*, group A *Streptococcus*

## Abstract

The recently observed increase in invasive *Streptococcus pyogenes* infections causes concern in Europe. However, conventional molecular typing methods lack discriminatory power to aid investigations of outbreaks caused by *S. pyogenes*. Therefore, there is an urgent need for high-resolution molecular typing methods to assess genetic relatedness between *S. pyogenes* isolates. In the current study, we aimed to develop a novel high-resolution core-genome multilocus sequence typing (cgMLST) scheme for *S. pyogenes* and compared its discriminatory power to conventional molecular typing methods. The cgMLST scheme was designed with the commercial Ridom SeqSphere+ software package. To define a cluster threshold, the scheme was evaluated using publicly available data from nine defined *S. pyogenes* outbreaks in the United Kingdom. The cgMLST scheme was then applied to 23 isolates from a suspected *S. pyogenes* outbreak and 117 *S*. *pyogenes* surveillance isolates both from the Netherlands. MLST and *emm*-typing results were used for comparison to cgMLST results. The allelic differences between isolates from defined outbreaks ranged between 6 and 31 for isolates with the same *emm-*type, resulting in a proposed cluster threshold of <5 allelic differences out of 1,095 target loci. Seven out of twenty-three (30%) isolates from the suspected outbreak had an allelic difference of <2, thereby identifying a potential cluster that could not be linked to other isolates. The proposed cgMLST scheme shows a higher discriminatory ability when compared to conventional typing methods. The rapid and simple analysis workflow allows for extended detection of clusters of potential outbreak isolates and surveillance and may facilitate the sharing of sequencing results between (inter)national laboratories.

## INTRODUCTION

*Streptococcus pyogenes* [group A *Streptococcus* (GAS) according to the Lancefield classification (1)] is a bacterium that causes a large range of diseases, varying from superficial skin diseases and pharyngitis to more severe diseases such as necrotizing fasciitis and is associated with significant morbidity and mortality (2). Although severe GAS infections occur sporadically, community outbreaks (OBs) of GAS have been frequently reported (3 – 5). In the Netherlands, a post-COVID-19 increase in invasive group A streptococcal disease (iGAS) was noted by the national surveillance program (6, 7). This trend is also observed in other European countries where recent reports show an increase in outbreaks of iGAS, especially among children (8).

Identification of an outbreak has traditionally been made based on the combination of molecular typing of isolates and the presence of an epidemiological link. Commonly used molecular typing methods to differentiate among *S. pyogenes* isolates include single-locus sequence typing of the *emm*-gene and a multilocus sequence typing (MLST) scheme published by Tewodros and Kronvall (9). Sequence analysis of 180 bp of the *emm*-gene, which encodes the M protein, has resulted in differentiation of more than 200 *S*. *pyogenes* M genotypes that are associated with varying levels of virulence (9). In MLST, nucleotides of seven housekeeping genes are used to analyze the genetic relationships of *S. pyogenes*, resulting in an allelic profile (sequence type or “ST”) (10). At least 1,367 different STs have been identified over the years (11). Although these conventional sequence-based typing methods are highly robust and the data achieved by different laboratories can be reliably compared using online databases, these methods may lack sufficient discriminatory power to distinguish different isolates within the same lineage that may be responsible for an outbreak (12). Therefore, there is a need for higher-resolution molecular typing methods to assess genetic relatedness between *S. pyogenes* strains.

Whole-genome sequencing (WGS) has the ability to produce more discriminatory power to differentiate isolates and evaluate outbreaks by including a higher number of target genes (13). Next-generation sequencing (NGS) has enabled the cost-effective implementation of WGS in the diagnostic laboratory. Genome similarities can be investigated by single-nucleotide polymorphism (SNP)-based mapping, in which sequence reads are compared to a reference genome to identify SNP variations and define clusters (14, 15). A disadvantage of this method is that it is difficult to standardize between laboratories due to differences in quality assurance criteria and reference genomes (16).

For several bacterial species, gene-by-gene-based approaches, such as whole-genome MLST (wgMLST) or core-genome MLST (cgMLST) schemes have been used for strain differentiation (17).

CgMLST schemes are developed using a fixed set of target genes (i.e., core genes) allocated throughout the genome that can be identified in most strains of a particular species (18). For different bacterial species, this technique proved to be a highly discriminative, efficient, and reliable tool for the differentiation of strains (17). Using a fixed set of target genes, a standardized comparison between laboratories can be achieved. A recent study by Friães et al. found that wg/cgMLST has a higher discriminatory power for the detection of *S. pyogenes* isolate variations than the conventional MLST schema and could, therefore, assist in further discriminating within STs (19). To our knowledge, there are no other studies available that used cgMLST for *S. pyogenes* typing. So far, cgMLST schemes for *S. pyogenes* strains using widely available analyzing software such as Ridom SeqSphere+ are lacking. In the current study, we aimed to develop such a novel cgMLST scheme using a local cluster and publicly available data sets.

## MATERIALS AND METHODS

### Development of the *S. pyogenes* cgMLST scheme

A novel cgMLST scheme was designed with the commercially available Ridom SeqSphere+ (version 8.3.5) software package (20). We used WGS data of 66 publicly available *S. pyogenes* genomes, comprising 38 different *emm*-types and 45 different sequence types, adopted from the NCBI RefSeq database (21) that was downloaded on 19 August 2022. A total of 1,095 common target genes within the genome of each strain were identified and used for developing the cgMLST scheme. The 66 genomes that were used represent a broad spectrum of S. *pyogenes* strains (Table S1).

### Evaluation of the *S. pyogenes* cgMLST scheme (Data set 1)

The cgMLST scheme was evaluated using Data set 1, which included publicly available data (52 isolates from nine different *S. pyogenes* outbreaks that were independently reported to Public Health England with available epidemiology data), which were documented in England (2010–2015) (22). Previous analyses using wg/cgMLST confirmed that these isolates were indeed part of an outbreak (19). These data have been used to determine a cluster threshold to accurately distinguish isolates from different clonal lineages. FastA-files were downloaded from an open data source (23) and processed further using Ridom SeqSphere+ (version 8.3.5) and PubMLST (24) for data analysis. For comparability, the same OB numbers as in the initial publication have been used (22).

### Application of the *S. pyogenes* cgMLST scheme (Data sets 2 and 3)

The cgMLST scheme was applied to isolates from a potential *S. pyogenes* outbreak and WGS-data from (un)related *S. pyogenes* isolates provided by the Netherlands Reference Laboratory for Bacterial Meningitis (NRLBM). Data set 2 consisted of 23 possible outbreak-related clinical *S. pyogenes* isolates collected between December 2021 and October 2022 at Leiden University Medical Center (LUMC), the Netherlands. The included *S. pyogenes* isolates were identified for clinical diagnostic purposes using matrix-assisted laser desorption/ionization- time of flight (MALDI-TOF) mass spectrometry (25) and Streptex agglutination tests, after overnight incubation of the clinical specimen on blood agar (BioMérieux SA). To examine whether the isolates were outbreak-related, the samples were analyzed by *emm*-typing, MLST, and cgMLST. For this, DNA was extracted and purified from cultured *S. pyogenes* strains using the QIAamp DNA Blood Mini Kit (QIAGEN Benelux BV). From the purified DNA extracts, NGS libraries were prepared with the Illumina DNA Prep (Illumina, San Diego, CA, USA), which was bidirectionally sequenced using the Illumina MiniSeq platform with 2 × 150 bp chemistry (Illumina, San Diego, CA, USA). FastQ-formatted sequences were extracted from the MiniSeq machine and processed further using Ridom SeqSphere+ and PubMLST (https//pubmlst.org) for data analysis.

The third data set (Data set 3) included WGS data of 117 samples provided by the NRLBM. Medical microbiology laboratories from the Netherlands send their *S. pyogenes* isolates cultured from a normally sterile site to the NRLBM for national surveillance purposes (*emm*-typing) (26). No detailed epidemiological information was available for these isolates. The included samples were collected between 2009 and 2022. FastA-files were provided by the NRLBM and processed at the LUMC for data analysis. All samples included in this study have been anonymized and are not traceable to individuals, omitting the need for approval by an ethical committee.

Distances between target genes (allelic differences) were presented using minimum-spanning trees. In the scheme, the minimum depth of coverage was 30 and minimum base Q values was set at 120. The number of contigs was <1,000 and contig N50 was >15,000. The assembler was Velvet 1.1.04. Genome size was 1.83 kb. The lowest percentage of included cgMLST targets was 99%. All missing loci were removed as a whole from the analysis. Standard MLST scheme (seven target genes) (10) and *emm*-typing (27, 28) were determined to compare with cgMLST results.

## RESULTS

### Conventional typing methods and cgMLST scheme

The core genome for the cgMLST scheme was defined as a standard of 1,095 target genes using the default setting of the cgMLST target definer in combination with one seed genome and 66 different S. *pyogenes* genomes (Table S1). Using conventional typing methods, 34 different sequence-types and 20 *emm-*types were identified in Data sets 1–3.

### Performance of cgMLST on publicly available WGS data

**Fig 1:**
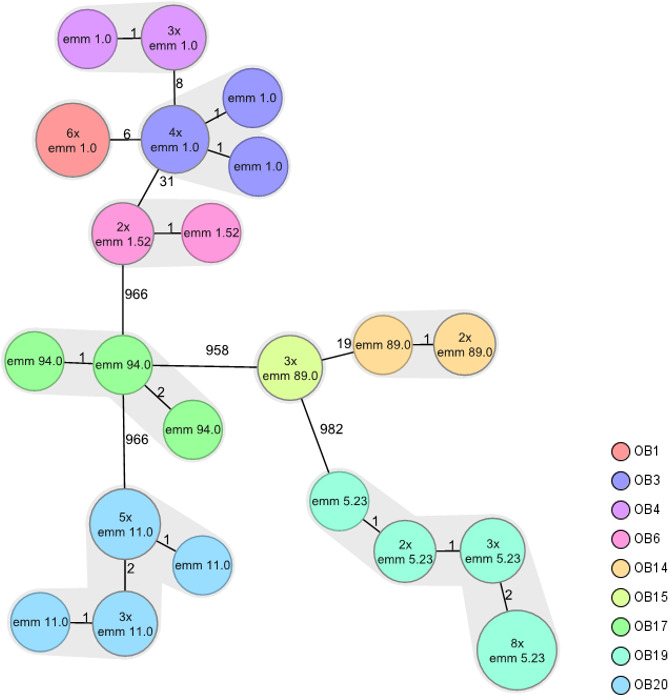
Minimum-spanning tree constructed using cgMLST profiles of 52 *S. pyogenes* isolates that were part of nine confirmed *S. pyogenes* outbreaks in England, UK (2010–2015). (i) OB = outbreak. (ii) The numbers at the connecting lines indicate the number of allelic differences between isolates. The numbers within the circles indicate the *emm*-type and the number of isolates with the same genetic profile (without allelic differences). The cluster threshold was set at <5 allelic differences.

The proposed scheme was applied to publicly available (outbreak) WGS data from previous publications. When applying the scheme to isolates from previously defined outbreaks, the maximum allelic difference between isolates within one outbreak was two. The allelic differences between isolates from different outbreaks ranged between 6 and 31 for isolates with the same *emm-*type and between 958 and 982 for outbreaks with a different *emm*-type (Fig. 1). Based on these results, we propose a cluster threshold of <5 allelic differences for the current cgMLST scheme.

### Performance of cgMLST with clinical isolates

**Fig 2:**
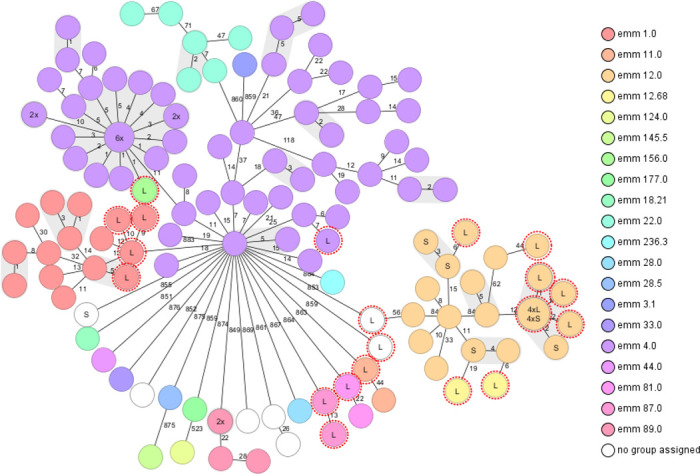
Minimum-spanning tree constructed using cgMLST profiles of 140 clinical isolates from the Netherlands. (i) OB = outbreak. (ii) Of the 140 profiles, 23 were derived from clinical isolates of patients and health-care workers from the Leiden University Medical Center, indicated with L and a red dotted line (Data set 2). The remaining 117 profiles were obtained from the NRLBM (Data set 3) and included nine profiles (S) of patient isolates with a geographic link to the Leiden region. The cluster threshold was set at <5; the gray shading indicates a cluster.

The developed cgMLST scheme was challenged with different strains from 16 different *emm*-types. Based on the previous analyses using outbreak data, the cluster threshold was defined at <5. The percentages of included cgMLST genes were >99%. The 23 isolates from the suspected outbreak (Data set 2) are shown in Fig. 2. Of those, seven isolates had an allelic difference of <2, thereby identifying a potential cluster. The cgMLST scheme was able to discriminate between isolates that belonged to the same ST or *emm*-types. From the NLRBM isolates (Data set 3), allelic differences between isolates belonging to the same *emm-*type ranged between 0 and 118. The number of isolates that are within potential clusters was 60, identifying 15 potential outbreak clusters.

## DISCUSSION

The proposed cgMLST scheme shows a higher discriminatory ability when compared to conventional typing methods. When using the cgMLST scheme on WGS data from previous publications containing *S. pyogenes* isolates from defined outbreaks, the maximum allelic difference between isolates from an outbreak was two, and the minimum allelic distance between isolates from different outbreaks was six. The proposed cluster definition for the cgMLST scheme is, therefore, <5 allelic differences. When the cgMLST scheme was applied to 140 clinical isolates from the Netherlands, 15 clusters could be identified based on the proposed cluster threshold. However, since no epidemiological data were available, it was not possible to define these clusters as outbreaks. Consistent across all isolates analyzed, cgMLST was able to identify allelic differences between isolates that had identical ST or *emm-*types, showing a higher discriminatory ability.

This study used a selection of WGS data (22) from isolates published in a previous study by Friães et al. (19), in which a different wg/cgMLST scheme for *S. pyogenes* was proposed. This group reports that their cgMLST scheme results were comparable to SNP-based methods, with a higher discriminatory power when compared to conventional typing methods. Our proposed cgMLST algorithm technique was developed with the commercially available Ridom SeqSphere+ software, whereas Friães et al. used chewBBACA (BSR-Based Allele Calling Algorithm) software. ChewBBACA software is freely available, but the software is command line-based, making it more complex to use compared to SeqSphere+ software. Friães et al. report a maximum link distance of six allelic differences within *S. pyogenes* outbreaks similar to our findings (Data set 1, maximum link distance two). Outbreaks containing isolates that were excluded based on wg/cgMLST results in the study by Friães et al. were not analyzed in our study.

CgMLST schemes for several other bacterial species have been established and evaluated. They were developed in recent years and can be used for the evaluation of strain differentiation and help to identify outbreaks. For example, in a recent study, cgMLST was reliably used for high-resolution typing of outbreaks with *Brucella* strains (29). For many bacterial species, commercially available software such as Ridom SeqSphere+ or BioNumerics published fixed cgMLST bacterial gene schemes for the standardization of WGS-based bacterial genotyping (30). For some of these species (including *S. pyogenes*), the maximum allelic distance or cluster type threshold within an outbreak is unknown. Previous studies have observed that allelic distances within outbreaks were less than 10 alleles for species such as *Klebsiella pneumoniae, Listeria monocytogenes, Mycobacterium tuberculosis,* and *Legionella pneumophila* (31 – 34). For *Staphylococcus aureus*, the cluster threshold distance is estimated to be 10–25 (35, 36). In the outbreak data used in our study (Fig. 1), the maximum distance between target genes within an outbreak was <6. We, therefore, propose a threshold of <5 to identify *S. pyogenes* isolates that are likely to belong to the same outbreak. For *Clostridioides difficile*, a similar low threshold (<6) is suggested to define the maximum distance between epidemiologically linked clusters (37).

The proposed cgMLST scheme for *S. pyogenes* has several advantages. It has the potential to enable widespread improvement of genomic surveillance and outbreak detection of *S. pyogenes*. The standardized analysis workflow of this cgMLST scheme is performed using easy-to-use software (Ridom SeqSphere+) and is made available for users of this software (https://www.cgmlst.org/ncs/schema/30585223/). Especially at a time when there is an increase in outbreaks of invasive GAS, and NGS becomes more widely available in many laboratories, a standardized cgMLST typing scheme with high discriminatory power is needed whereby the results can be easily exchanged between (inter)national laboratories. Of note, Ridom SeqSphere + is not free of charge and although it can be used for MLST and genomic antimicrobial resistance determination, *emm*-typing is currently not possible with this software. Future updates to include *emm*-typing would be a meaningful update.

The current study also has some limitations. Most importantly, detailed epidemiological information was lacking for most isolates (Data sets 2 and 3), which complicates reliably identifying outbreaks. Second, the cgMLST technique by design only detects allele variations that have been previously defined as the core genome (18). Techniques such as wgMLST or SNP include more coverage of the complete genome (including the accessory genome) and potentially achieve a higher resolution, which further increases discriminatory power. For example, in a recent article from the Netherlands [currently available in preprint (26)], whole-genome sequence analysis of *emm*4 isolates identified a novel S. *pyogenes* lineage, accounting for 85% of the *emm*4 invasive S. *pyogenes* cases in 2022. However, for reproducibility and to compare results between laboratories, it is desirable to use a standardized cgMLST method with easy-to-use software. Due to the lack of available studies with epidemiological data, the proposed cluster threshold of <5 allelic differences is based on a single outbreak study only, and more data are necessary to support this.

In conclusion, the robustness of this cgMLST scheme that is made publicly available via Ridom, in combination with easy-to-use software, enables widespread improvement of genomic surveillance of *S. pyogenes*, allowing increased detection of transmission and highlighting opportunities for intervention.

## Data Availability

Raw sequence reads for Data set 1 can be found in the Sequence Read Archive (SRA) under project number PRJEB49967 (38). Raw sequence reads for Data set 2 have been deposited in the SRA under project accession number PRJNA966900 (39). Raw sequence data for Data set 3 have been deposited in the SRA under project accession number PRJNA967239 (40).

## SUPPLEMENTAL MATERIAL

Table S1 jcm.00558-23-s0001.docx List of Emm-types and STs from the 66 penetration query genomes

ASM does not own the copyrights to Supplemental Material that may be linked to, or accessed through, an article. The authors have granted ASM a non-exclusive, world-wide license to publish the Supplemental Material files. Please contact the corresponding author directly for reuse.
