# Supplementary material for: A core-genome multilocus sequence typing scheme for the detection of genetically related Streptococcus pyogenes clusters
Source: J Clin Microbiol. 2023 Oct 10;61(11):e00558-23. doi: 10.1128/jcm.00558-23 (PMC10662357; doi:10.1128/jcm.00558-23)
Supplement: Table S1 — List of Emm-types and STs from the 66 penetration query genomes [file jcm.00558-23-s0001.docx]

**SUPPLEMENTAL MATERIAL**

| **Accessionnumber** | ***Emm*-type** | **Sequence Type** |
| --- | --- | --- |
| NC_002737.2 | 1.0 | 28 |
| NC_003485.1 | 18.19 | 42 |
| NC_004606.1 | 3.1 | 15 |
| NC_006086.1 | 6.4 | 382 |
| NC_009332.1 | 5.0 | 99 |
| NC_011375.1 | 49.1 | 1073 |
| NC_017596.1 | 53.0 | 11 |
| NC_017040.1 | 59.0 | 172 |
| NC_020540.2 | 1.0 | 28 |
| NC_018936.1 | 1.0 | 28 |
| NC_021807.1 | 14.3 | 84 |
| NZ_HG316453.1 | 89.0 | 101 |
| CP007024.1 | 44.0 | 178 |
| NZ_CP007041.1 | 3.2 | 406 |
| NZ_CP008926.1 | 80.1 | 8 |
| NZ_CP008695.1 | 23.0 | 160 |
| NZ_CP007240.1 | 83.1 | 5 |
| NZ_CP009612.1 | 12.0 | 36 |
| AP014572.1 | 1.0 | 28 |
| NZ_LN831034.1 | 1.0 | 28 |
| NZ_AP012335.1 | 6.52 | 37 |
| NZ_CP011415.1 | 6.52 | 37 |
| NZ_CP012045.1 | 1.0 | 28 |
| NZ_CP010449.1 | 114.0 | 188 |
| NZ_CP014139.1 | 28.0 | 52 |
| NZ_CP014027.2 | 1.0 | 28 |
| NZ_CP015238.2 | 71.0 | 318 |
| NZ_AP017629.1 | 89.0 | 101 |
| NZ_CP022354.1 | 111.2 | 1065 |
| NZ_AP018337.1 | 3.95 | 15 |
| NZ_CP021640.1 | 179.0 | 619 |
| NZ_CP028148.1 | 12.0 | 36 |
| NZ_CP028140.1 | 74.0 | 120 |
| NZ_LS483356.1 | 51.0 | 158 |
| NZ_LS483521.1 | 203.4 | 75 |
| NZ_LS483335.1 | 12.0 | 1312 |
| NZ_CP027771.1 | 81.0 | 330 |
| NZ_CP031770.1 | 4.0 | 39 |
| NZ_CP033815.1 | 3.60 | 315 |
| NZ_CP033907.1 | 4.0 | 39 |
| NZ_LR031521.1 | 1.0 | 28 |
| LR134272.1 | 80.0 | 538 |
| NZ_LR134284.1 | 47.1 | 1271 |
| NZ_LR134314.1 | 6.52 | 37 |
| NZ_LR130238.1 | 1.0 | 28 |
| NZ_CP032666.1 | 28.0 | 52 |
| NZ_LR130240.1 | 28.0 | 52 |
| NZ_CP033621.1 | 75.0 | 150 |
| NZ_CP031633.1 | 28.0 | 456 |
| NZ_CP031628.1 | 28.0 | 52 |
| NZ_CP031620.1 | 28.0 | 52 |
| NZ_CP031619.1 | 28.0 | 458 |
| NZ_CP035453.1 | 100.0 | 119 |
| NZ_CP035452.1 | 123.0 | 325 |
| NZ_CP035440.1 | 124.0 | 199 |
| NZ_CP035430.1 | 55.0 | 100 |
| NZ_CP035433.1 | 65.0 | 716 |
| NZ_CP035446.1 | 68.2 | 989 |
| NZ_CP065927.1 | 9.0 | 603 |
| NZ_CP055246.1 | 4.0 | 39 |
| NZ_CP060649.1 | 118.0 | 167 |
| NZ_CP060640.1 | 84.0 | 1056 |
| NZ_CP072523.1 | 3.1 | 15 |
| NZ_CP064364.1 | 1.0 | 28 |
| NZ_CP066541.1 | 92.0 | 82 |
| NZ_CP097251.1 | 22.0 | 46 |

Table S1. *List of Emm-types and STs from the 66 penetration query genomes, which were used to identify the core genes in our CgMLST scheme*
